# PRPF8 is important for BRCA1-mediated homologous recombination

**DOI:** 10.18632/oncotarget.21555

**Published:** 2017-10-06

**Authors:** David O. Onyango, Gabriella Lee, Jeremy M. Stark

**Affiliations:** ^1^ Department of Cancer Genetics and Epigenetics, Beckman Research Institute of the City of Hope, Duarte, CA, USA; ^2^ Irell and Manella Graduate School of Biological Sciences, Beckman Research Institute of the City of Hope, Duarte, CA, USA

**Keywords:** PRPF8, BRCA1, homologous recombination, interchromatin granules, single strand annealing

## Abstract

Disruption of RNA splicing causes genome instability, which could contribute to cancer etiology. Furthermore, RNA splicing is an emerging anti-cancer target. Thus, we have evaluated the influence of the spliceosome factor PRPF8 and the splicing inhibitor Pladienolide B (PlaB) on homologous recombination (HR). We find that PRPF8 depletion and PlaB treatment cause a specific defect in homology-directed repair (HDR), and single strand annealing (SSA), which share end resection as a common intermediate, and BRCA1 as a required factor. Furthermore, PRPF8 depletion and PlaB treatment cause reduced end resection detected as chromatin-bound RPA, BRCA1 foci in response to damage, and histone acetylation marks that are associated with BRCA1-mediated HR. We also identified distinctions between PlaB and PRPF8 depletion, in that PlaB also reduces 53BP1 foci, and BRCA1 expression. Furthermore loss of 53BP1, which rescues SSA in BRCA1 depleted cells, and partially rescues SSA in PRPF8 depleted cells, has no effect on SSA in PlaB treated cells. Finally, while PRPF8 depletion has no obvious effect on the integrity of interchromatin granules, PlaB disrupts these structures. These findings indicate that PRPF8 is important for BRCA1-mediated HR, whereas PlaB also has a more general effect on the DNA damage response and nuclear organization.

## INTRODUCTION

Factors involved in RNA splicing have been linked to tumor suppression, and are also emerging as cancer therapeutic targets. In particular, recurrent mutations in splicing factors have been found in myeloid malignancies, including mutations in PRPF8 and SF3B1 [1, 2]. SF3B1, a component of the U2 snRNP, is also the target of antineoplastic agents, such as Pladienolide B (PlaB) [2-4]. The role of splicing factors in tumor suppression and as therapeutic targets likely includes their central role in shaping the transcriptome, and hence proper regulation of gene expression [1, 5]. However, disruption of RNA splicing factors have also been shown to cause genome instability [6, 7], which is both a hallmark of cancer, as well as a contributor to the therapeutic response to clastogenic anti-cancer agents [8]. Thus, understanding the links between RNA splicing and genome stability will provide insight into cancer etiology and development of therapeutic targets.

Inhibition of RNA splicing appears to cause genome instability by at least two major mechanisms. First, defects in RNA splicing can lead to elevated RNA/DNA hybrids at transcription units (i.e., R-loops), which are prone to nucleolytic cleavage, as well as collisions with DNA replication forks, causing chromosomal breaks [9]. Second, RNA splicing appears important for homologous recombination (HR) repair of chromosomal breaks, which is critical for genome stability and tumor suppression. HR is composed of two major pathways: homology-directed repair (HDR), which involves RAD51-mediated strand invasion to template nascent DNA synthesis that bridges the break, and single strand annealing (SSA), which uses synapsis of homology flanking a chromosomal break to mend the DNA lesion [10, 11]. These two pathways of HR are initiated by end resection to generate 3’ ssDNA, and share a common requirement for the BRCA1 tumor suppressor gene [10, 11]. The requirement of RNA splicing for HR has been revealed in RNAi screening studies. Specifically, screens for factors important for the HDR sub-type of HR identified several RNA processing factors, such as RBMX and the U2 snRNP complex [12, 13].

We have sought to further examine the links between RNA splicing and HR, building on a recent report from our lab that the spliceosome factor XAB2 promotes HDR and SSA, end resection, and focal accumulation of BRCA1 at chromosomal breaks [14]. These functions of XAB2 correlated with its ability to form a complex with ISY1 and PRP19, which showed similar roles in HR [14]. Other reports have also demonstrated that PRP19 is important for the DNA damage response [15-18]. However, it is unclear whether the roles of these factors are linked to RNA splicing functions. Similarly, it is unclear if disrupting RNA splicing using different approaches causes distinct effects on the DNA damage response. Thus, given that XAB2 has a conserved association with the central splicing factor PRPF8 [19], we examined the role of this factor in HR and the DNA damage response. Furthermore, we have compared effects of PRPF8 depletion with PlaB treatment.

## RESULTS

### Depletion of PRPF8 and treatment with a small molecule spliceosome inhibitor (PlaB) disrupts HDR, SSA, and end resection

**Figure 1:**
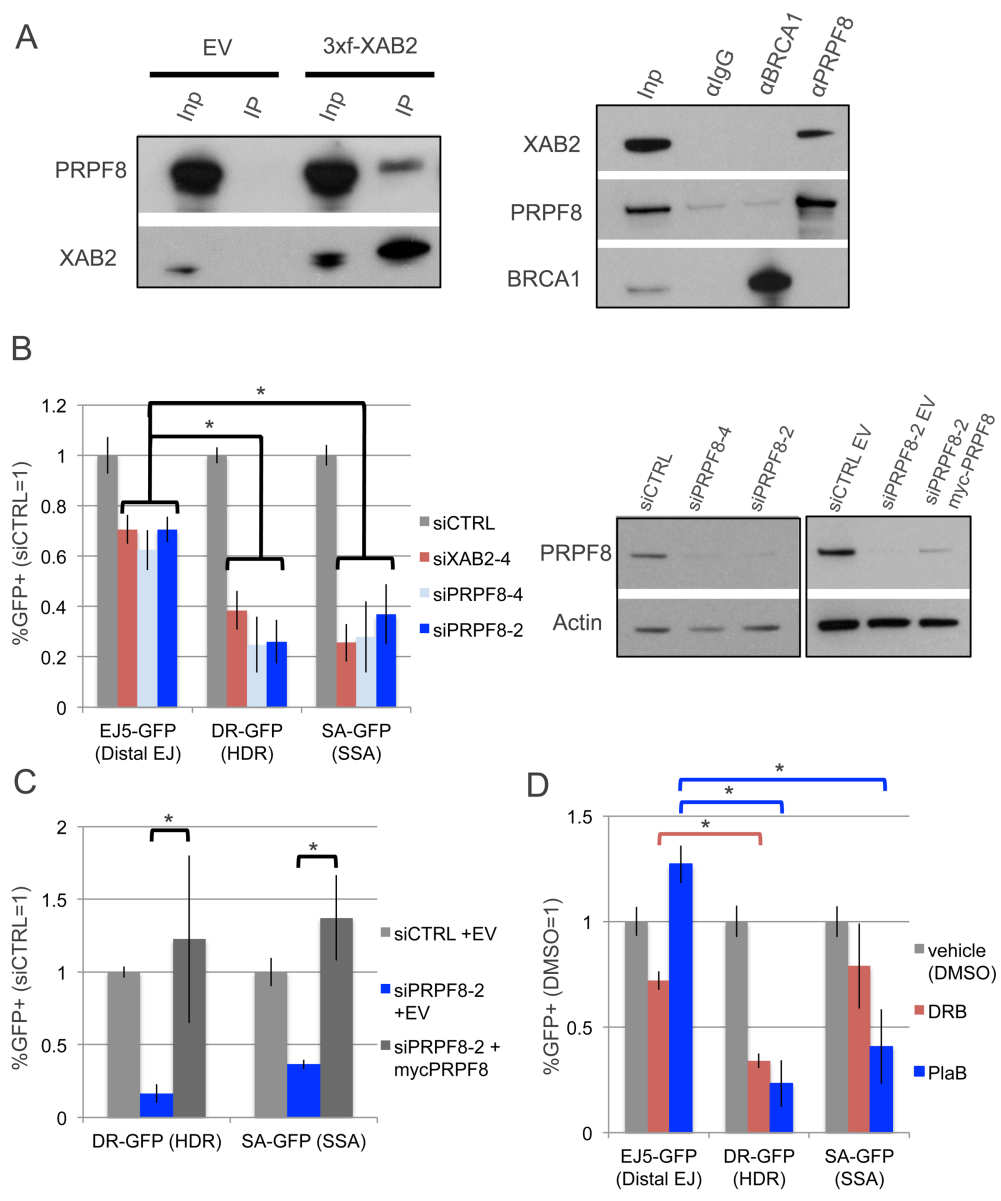
Depletion of PRPF8 and PlaB treatment have a similar effect on HR as XAB2 depletion **A.** PRPF8 forms a complex with XAB2. Shown are immunoblot signals for PRPF8 and XAB2 from Flag-immunoprecipitates of U2OS cells transfected with a 3xFlag-XAB2 (3xf-XAB2) expression vector or empty vector (EV). Also shown are immunoblot signals for XAB2, PRPF8, and BRCA1 for U2OS cells immunoprecipitated with antibodies against (α) PRPF8, BRCA1, or control IgG. Also shown are input (Inp) samples used for the immunoprecipitates (IP). **B.** PRPF8 is important for HDR and SSA to a greater degree than EJ. U2OS cell lines with GFP-based DSB reporter assays were pretreated with siRNAs targeting *PRPF8* (siPRPF8-2, siPRPF8-4), *XAB2* (siXAB2-4), or a non-targeting control (siCTRL), prior to expression of I-SceI and analysis by flow cytometry. Shown are GFP+ frequencies normalized to the mean of parallel siCTRL-treated samples. *P ≤ 0.0004, *N* = 6. Also shown are immunoblot signals confirming depletion of PRPF8 by siPRPF8-2 and siPRPF8-4, as well as expression of myc-PRPF8, with an Actin loading control. The panel on the left (comparison of PRPF8 levels in cells treated with siRNA) was an independent experiment and analyzed on a separate immunoblot from the panel on the right (comparison of PRPF8 levels in cells treated with siRNA and transfected with myc-PRPF8 or EV). **C.** Transient expression of PRPF8 rescues HDR and SSA in cells treated with siPRPF8-2. Reporter assays were performed as in B, except an expression vector for myc-PRPF8 with silent mutations at the siPRPF8-2 targeting sequence (expression shown in B), or EV, were included in the transfection with the I-SceI expression vector. **D.** PlaB treatment causes a decrease in HDR and SSA, but not EJ, whereas DRB causes a greater defect on HDR *vs*. either EJ or SSA. U2OS reporter cell lines were treated with non-targeting RNA (siCTRL) as in A, and transfected with an inducible form of I-SceI (I-SceI-GR). The day after transfection, cells were pre-treated (2hr) with PlaB, DRB, or vehicle (DMSO), and then treated overnight with the same small molecules, but also including the I-SceI-GR inducing agent (TA). The cells were cultured for an additional day prior to flow cytometry analysis. Shown are GFP+ frequencies normalized to parallel DMSO-treated wells. *P < 0.0001. *N* = 6 for DR-GFP and EJ5-GFP. *N* = 12 for SA-GFP.

We sought to examine the influence of PRPF8 on chromosomal double strand break (DSB) repair, based on findings that PRPF8/PRP8 forms a conserved complex with XAB2/SYF1 [19], which has been shown to be important for the end resection step of homologous recombination (HR) in human cells [14]. First, we examined whether PRPF8 and XAB2 form a complex in the human osteosarcoma U2OS cell line, by performing co-IP analysis. We found that IP samples of 3xFlag-immunotagged XAB2 contain PRPF8, and conversely IP samples of PRPF8 contain XAB2 (Figure1A). Notably, while an association of PRPF8 with the HR factor BRCA1 has been previously reported [20], we were unable to detect such a complex using the same buffer conditions (Figure1A), but perhaps these factors form a weak association that we could not detect. In any case, these findings are consistent with the conserved association XAB2 and PRPF8.

Thus, we next tested whether PRPF8 and XAB2 have a similar influence on DSB repair, using a series of chromosomal reporter assays integrated into U2OS cells [21]. Each of these reporters is designed such that repair of an I-SceI-induced DSB by a specific pathway restores GFP expression, which can be quantified by flow cytometry (Supplementary Figure 1). We examined three reporters: EJ5-GFP to examine end-joining (EJ) repair between two tandem DSBs, DR-GFP for HDR, and SA-GFP for SSA (Supplementary Figure 1). Using two independent siRNAs targeting *PRPF8* (siPRPF8-2, siPRPF8-4), as well as a previously described siRNA targeting *XAB2* (siXAB2-4), we found that PRPF8 and XAB2 depletion cause a significantly greater decrease in the frequency of HDR and SSA, compared to EJ (Figure 1B). We also found that transient expression of a myc-tagged siRNA-resistant PRPF8 rescued the HDR and SSA defects caused by siPRPF8-2 treatment (Figure 1B, 1C). We also confirmed expression of myc-PRPF8 by immunoblotting analysis (Figure 1B). The apparently lower immunoblotting signal for myc-PRPF8 in this experiment, compared to endogenous PRPF8 (Figure 1B), likely reflects the limitations of transfection efficiency (i.e., only a fraction of cells are transfected).

Given that PRPF8 is a central factor in the RNA spliceosome [19, 22], we also wanted to compare effects of PRPF8-depletion with disruption of RNA splicing, using the small molecule Pladienolide B (PlaB), which targets SF3B1 [2-4]. For an additional comparison, we examined 5,6-Dichlorobenzimidazole 1-β-D-ribofuranoside (DRB), which inhibits RNA polymerase II elongation [23]. We included DRB in this analysis, because disruption of RNA splicing has been associated with reduced levels of an elongation form of RNA polymerase II (i.e., phosphorylated in the serine 2 position of the C-terminal domain, POL2-S2P) [24]. For these small molecule treatments, we used an inducible form of I-SceI (I-SceI-GR), in which I-SceI is fused to a version of the ligand binding domain of the glucocorticoid receptor (GR) [25]. Thus, I-SceI-GR is restricted from the nucleus unless the inducing ligand (triamcinolone acetonide, TA) is added to the media [25]. Specifically, we transfected cells with an expression vector for I-SceI-GR, and the next day pre-treated the cells with PlaB, DRB, or vehicle (DMSO), followed by an overnight treatment that included the inducing agent for I-SceI-GR (i.e., TA), and cells were cultured an additional day before the analysis. We found that similar to PRPF8 depletion, PlaB treatment caused a marked reduction in HDR and SSA, compared to EJ (Figure 1D). In contrast, DRB treatment caused a reduction in HDR compared to EJ, whereas the reduction in SSA was not statistically different from the effect on EJ (Figure 1D). These findings indicate that PRPF8 depletion and PlaB treatment each cause a defect in HDR and SSA. In contrast, DRB treatment had a more specific effect on HDR, which may reflect a disruption of RAD51 recombinase function, which is required for HDR, but not SSA [26]. This possibility is consistent with a prior report that DRB treatment inhibits RAD51 association with chromosomal breaks, as measured by chromatin immunoprecipitation at DSBs induced by the AsiSI endonuclease [27].

**Figure 2:**
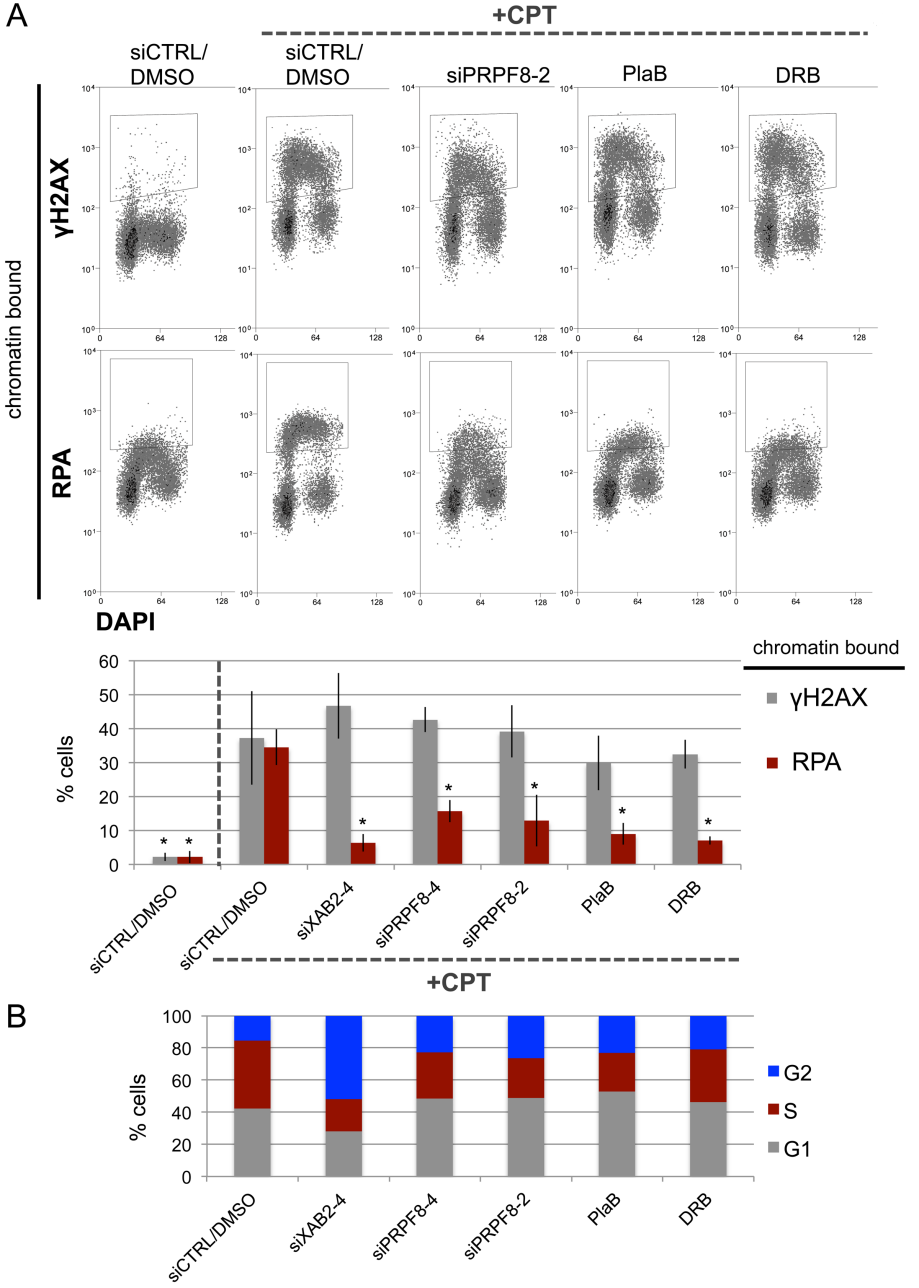
PRPF8 depletion and PlaB treatment cause a reduction end resection as detected by damage-induced chromatin bound RPA **A.** Cells were transfected with the siRNAs shown and cultured for 3 days prior to treatment with CPT (1 hr). For the small molecule treatments, cells were treated with siCTRL for consistency, and pre-treated with PlaB or DRB for 7 hours, prior to treatment with these small molecules and CPT (1hr). Cells were exposed to mild detergent extraction prior to fixation and staining with RPA34 or γH2AX, with DAPI counterstain. Shown are representative flow cytometry plots, as well as the percentage of cells showing detergent resistant (i.e., chromatin bound) RPA34 or γH2AX staining. *distinct from siCTRL/DMSO w/ CPT, *P* ≤ 0.0004. Targeting siRNAs *N* = 3, PlaB *N* = 5, DRB *N* = 4, siCTRL/DMSO w/ and w/o CPT *N* = 8 (higher N because a siCTRL/DMSO control was included each set of experiments). **B.** The treatments shown in A do not cause an increase in G1 phase cells. Cells were treated with siRNA as in A, and PlaB or DRB for 7.5 hrs (to maintain a total 8 hr treatment), followed by 30 minutes of pulse labeling with BrdU. Cells were stained for BrdU and counter stained with PI to determine the percentage of cells in G1, S, or G2, as shown. Shown are the mean values of *N* = 4 treatments, except siCTRL *N* = 8.

The initiating step that is shared between HDR and SSA is end resection to generate 3’ ssDNA that reveals the homology used during these repair events [10, 11, 28]. For a measure of end resection, we performed a flow cytometry based assay, involving detection of chromatin bound (i.e., extraction resistant) staining of the ssDNA binding protein RPA [29]. As previously described [29], treating U2OS cells with the topoisomerase I poison camptothecin (CPT) causes a significant induction of chromatin-bound RPA, as well as chromosomal breaks marked by γH2AX (Figure 2A). However, cells treated with siXAB2, siPRPF8-2, siPRPF8-4 prior to CPT treatment showed a significant reduction in cells with chromatin bound RPA, but not γH2AX (Figure 2A). Similarly, cells pre-treated with PlaB or DRB, and then co-treated with CPT, showed a significant reduction in cells with chromatin bound RPA, but not γH2AX (Figure 2A). Since end resection is suppressed in G1 cells [28], we examined whether siXAB2, siPRPF8, PlaB, or DRB treatment affected cell cycle profiles (BrdU / Propidium Iodide labeling), using the same treatment protocol (i.e., total timing and concentration) as for the end resection analysis, and found that none caused an increase in G1 phase cells (Figure 2B). These findings indicate that depletion of PRPF8, as well as treating cells with inhibitors of the spliceosome (PlaB) or transcription elongation (DRB) cause a defect in RPA localization to CPT-induced damage, which is a measure of end resection.

### PRPF8 depletion affects BRCA1 function during HR, whereas PlaB treatment also appears to have more general effects on the DNA damage response

**Figure 3:**
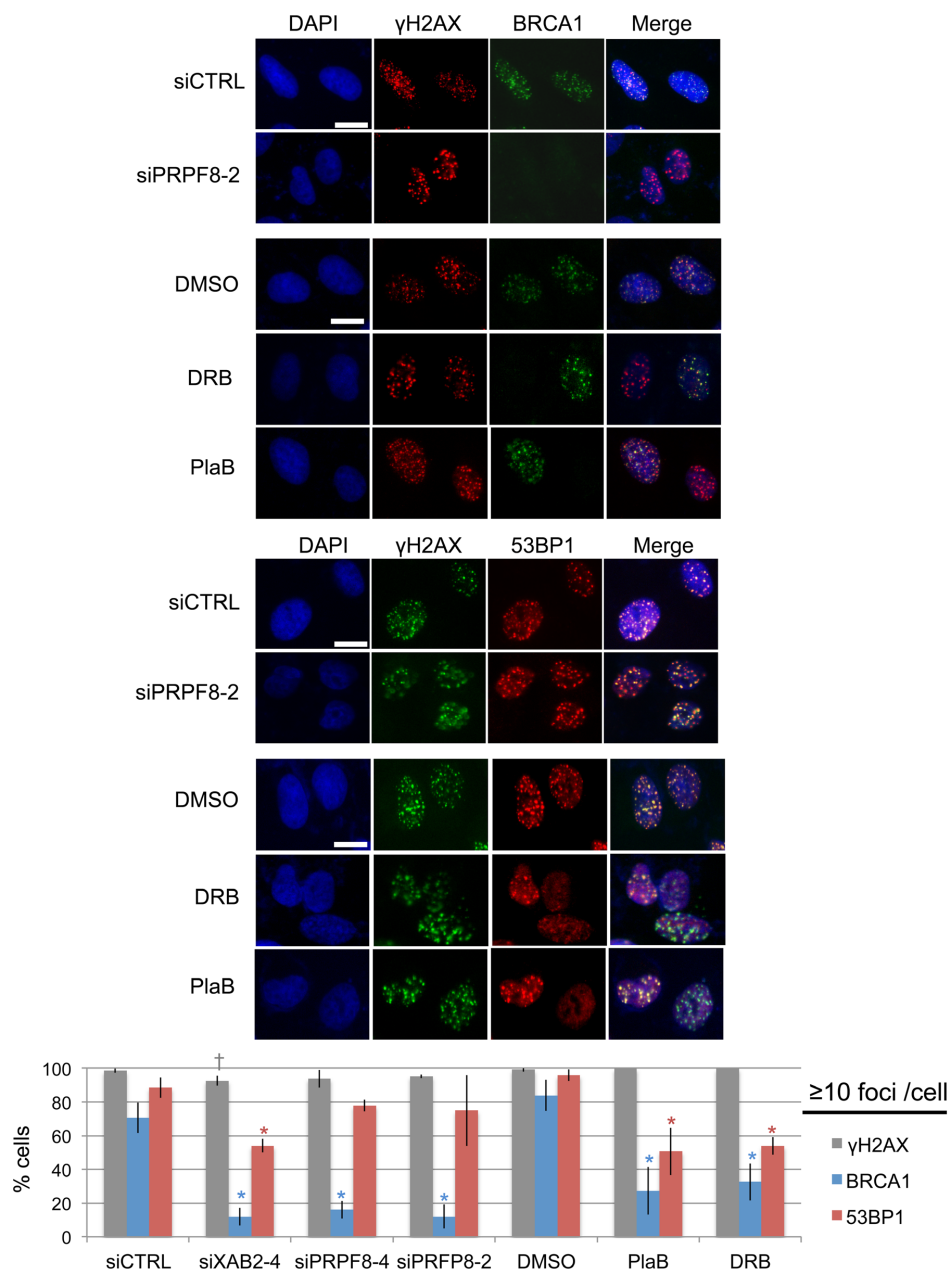
Depletion of PRPF8 causes a decrease in BRCA1 foci, whereas PlaB treatment causes a decrease in both BRCA1 and 53BP1 foci Cells were transfected with the siRNAs shown and cultured for 3 days prior to IR treatment (10 Gy) and 6 hr recovery. For small molecule treatments, cells were transfected with siCTRL for consistency, pre-treated with PlaB, DRB, or vehicle (DMSO) (2hrs), IR treatment (10 Gy) and 6 hr recovery in the presence of the small molecules or vehicle. Notably, the total treatment timing for these experiments is the same as experiments in Figure2 Shown are representative images of BRCA1, 53BP1, and γH2AX staining, for such treatments (scale bar = 10 μm). Shown is the percentage cells showing ≥10 foci for each marker shown distinct from siCTRL: *P ≤ 0.0042, †*P* = 0.034. *N* = 3, 50 cells analyzed per experiment.

We then examined the effects of these treatments on recruitment of key DNA damage response factors that are important for regulation of homologous recombination. For this, we examined ionizing radiation induced foci (i.e., cells with ≥10 foci) of BRCA1, 53BP1, and γH2AX. We examined BRCA1, since this factor is critical for HDR and SSA [10, 11, 28]. For an important contrast, we examined 53BP1, which inhibits homologous recombination, particularly in BRCA1-deficient cells [14, 30]. 53BP1 also shares with BRCA1 a common requirement for RNF8-mediated ubiquitin signaling pathway for foci formation [31-33]. From this analysis, we found that siPRPF8 treatment caused a marked reduction in BRCA1 foci, but not 53BP1 foci, similar to findings with siXAB2 treatment (Figure 3) [14]. In contrast, PlaB and DRB treatment caused a marked reduction in both BRCA1 and 53BP1 foci, although BRCA1 foci were disrupted to a greater degree (Figure 3). None of the treatments caused a substantial decrease in γH2AX foci (Figure 3). The finding that PlaB causes a reduction in both BRCA1 and 53BP1 foci was also observed in a recent report [34]. These results indicate that PRPF8 is important for BRCA1 foci formation, whereas PlaB and DRB treatment appear to cause more general defects in the DNA damage response.

**Figure 4:**
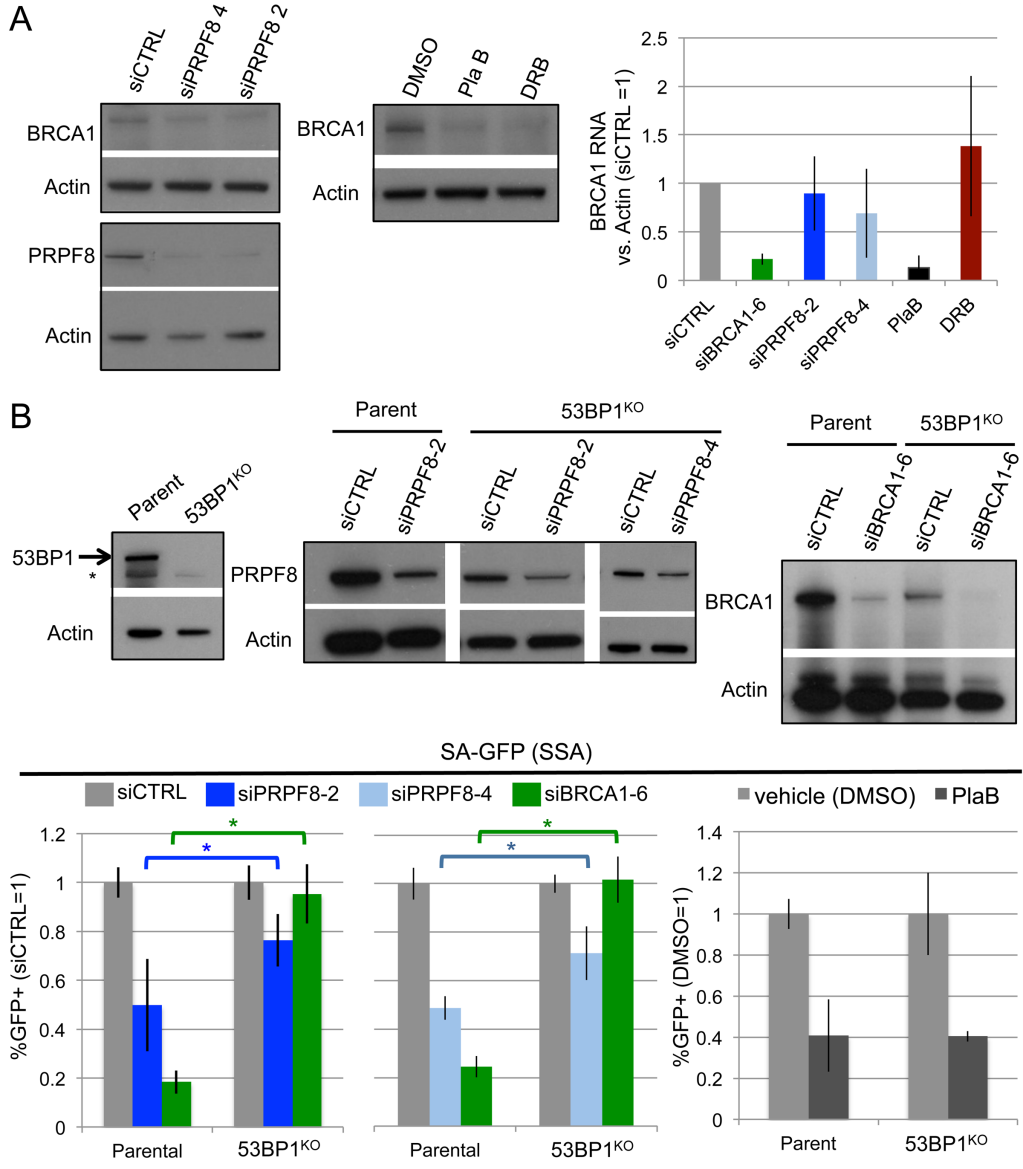
Effects of PRPF8 and PlaB on BRCA1 levels and SSA in 53BP1-deficient cells **A.** PlaB treatment causes a marked reduction in both BRCA1 protein and RNA. Cells were transfected with siRNA and cultured 3 days, and PlaB and DRB treatments were for 16 hr, prior to protein or RNA extraction, which were probed for BRCA1 and Actin by immunoblotting and qRT-PCR, respectively. **B.** Loss of 53BP1 partially suppresses the SSA defect caused by PRPF8 depletion, but not PlaB treatment. Using Cas9, a derivative of the SA-GFP cell line with loss of 53BP1 (53BP1^KO^ line) was generated, and reporter assays were performed as in Figure 1B and 1D. Shown are GFP+ frequencies normalized to parallel siCTRL or siCTRL/DMSO treated wells. Also shown is immunoblotting confirmation of depletion of BRCA1 and PRPF8 by the respective siRNAs, and loss of 53BP1 in the 53BP1^KO^ line. siPRPF8-2 graph, *P < 0.0001, parental line *N* = 15, 53BP1^KO^ *N* = 12. siPRPF8-4 graph *P = 0.001, *N* = 6. PlaB graph, parental line *N* = 12, 53BP1^KO^ *N* = 9.

Given the above effects on BRCA1 foci, we also examined effects of these treatments on BRCA1 levels, both protein and RNA (Figure 4A). For RNA analysis, BRCA1 transcript levels were normalized to Actin, using equivalent amounts of RNA extracted from cells. We found that siPRPF8 treatment caused a modest decrease in BRCA1 protein, but no obvious effects on its RNA levels, each compared to Actin. In contrast, PlaB treatment caused a substantial reduction in BRCA1 protein and RNA, which is consistent with a recent study [34]. DRB also caused a reduction in BRCA1 protein, without having an obvious effect on its relative RNA level, as compared to Actin. Notably, since DRB inhibits nascent RNA synthesis, the DRB experiment likely measures the relative stability, rather than synthesis, of BRCA1 and Actin transcripts.

We then tested a possible genetic connection between PRPF8 and BRCA1 function during HR. Namely, depletion of 53BP1 has been shown to suppress the HR defect, including the SSA defect, caused by BRCA1 loss [14, 30, 35]. Thus, we considered that 53BP1 loss might also suppress the SSA defect caused by PRPF8 depletion. For this, we generated a 53BP1^KO^ cell line with Cas9, using the U2OS SA-GFP reporter cell line (Figure 4B). We used the SA-GFP reporter cell line to disrupt 53BP1 so that the reporter assay would be isogenic between the parental and 53BP1^KO^ cell lines. We then examined the effect of BRCA1 depletion (siBRCA1-6 treatment), siPRPF8-2, and siPRPF8-4 treatment on the frequency of SSA in the parental and 53BP1^KO^ cell line. As expected, we found that siBRCA1-6 treatment caused a substantial defect in SSA in the parental line, but not the 53BP1^KO^ cell line (Figure 4B). We also found that siPRPF8-2, and siPRPF8-4 treatment caused an SSA defect in parental line, whereas in the 53BP1^KO^ cell line, the fold-effect was significantly diminished (Figure 4B). These findings indicate that the SSA defect caused by PRPF8 depletion can be partially suppressed by loss of 53BP1. We also examined PlaB treatment, and found that the SSA defect was not distinct between the parental and in the 53BP1^KO^ cell line (Figure 4B). These findings support the notion that PRPF8 functions during HR at least in part *via* facilitating BRCA1 function, whereas the effect of PlaB treatment on HR cannot be explained only as a loss of BRCA1 function. Furthermore, this distinction between PRPF8 and PlaB is consistent with our finding that PlaB treatment also causes a defect in 53BP1 foci.

### PRPF8 depletion and PlaB treatment have distinct effects on chromatin marks associated with homologous recombination and transcription unit function

**Figure 5:**
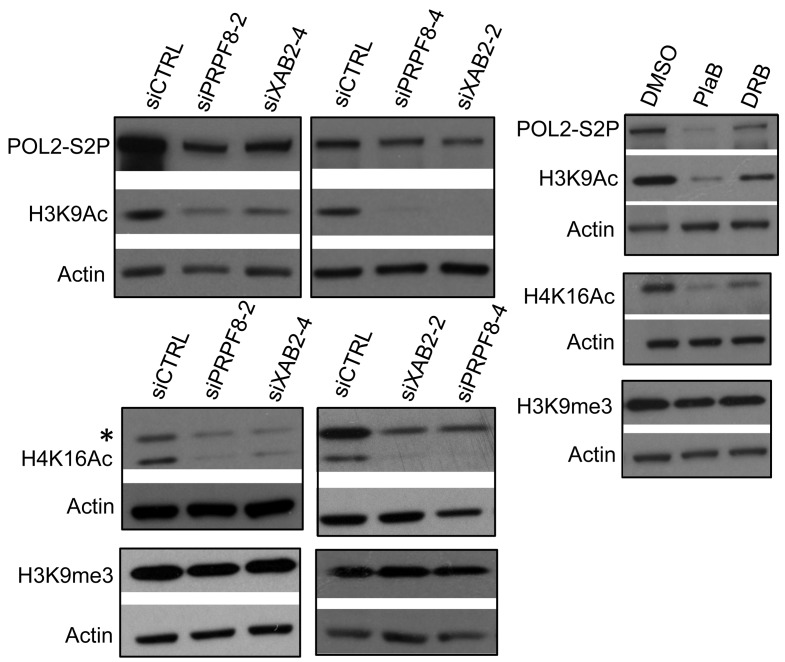
Both PRPF8 depletion and PlaB treatment cause a reduction in H3K9Ac and H4K16Ac, and the latter causes a marked reduction in POL2-S2P Cells were treated with siRNAs, PlaB, and DRB as in Figure 3, and chromatin was extracted for immunoblotting analysis of H3K9Ac, H4K16Ac, H3K9me3, POL2-S2P, and Actin. Shown are representative immunoblots from this analysis.

Given the above distinctions between PRPF8 depletion and PlaB treatment on the severity and relative specificity of effects on the DNA damage response, we then examined other aspects of chromatin signaling associated with HR and transcription unit function. To begin with, we examined histone acetylation levels, which have been linked to BRCA1 function and HR proficiency [27, 36, 37]. XAB2 depletion was previously shown to cause a decrease in two such histone acetylation marks (H3K9Ac and H4K16Ac) [14], which we have repeated here (Figure 5). Consistent with these findings with XAB2, we found that treatment with siPRPF8, PlaB, and DRB each caused a reduction in H3K9Ac and H4K16Ac, but not H3K9me3 (Figure 5). In addition, we examined global levels of POL2-S2P, since sites of active transcription marked by POL2-S2P have been shown to correlate with HR proficiency [27], and because splicing inhibition has recently been shown to cause a reduction in POL2-S2P [24]. We found that inhibition of transcription elongation by DRB treatment causes a substantial reduction in POL2-S2P, as did PlaB treatment (Figure 5). In contrast, siPRPF8 and siXAB2 treatment only caused a modest reduction in POL2-S2P (Figure 5). These findings indicate that a reduction in H3K9Ac and H4K16Ac levels are common for PRPF8 depletion and PlaB treatment, whereas PlaB has a greater effect on POL2-S2P levels than PRPF8 depletion.

**Figure 6:**
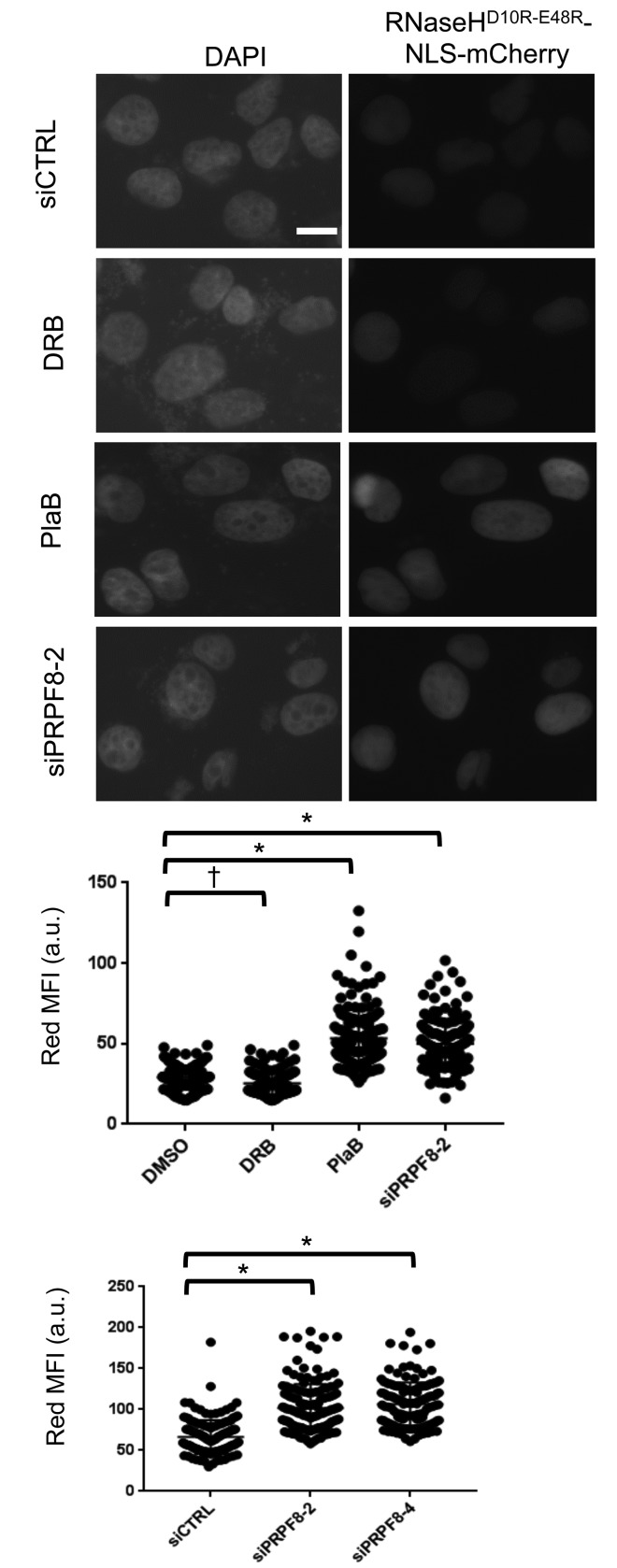
Both PRPF8 depletion and PlaB treatment cause an increase in chromatin bound RNaseH^D10R-E48R^, which is consistent with an accumulation of R-loops A U2OS cell line was generated with DOX-inducible expression of RNaseH^D10R-E48R^ fused to a nuclear localization signal and mCherry fluorescent protein. Cells were transfected with siRNA and two days later, DOX was added for 24 hr. For small molecule treatment, cells were treated with siCTRL for consistency, and DOX was added 6 hr prior to a 18 hr co-treatment with DOX and PlaB, DRB, or vehicle (DMSO). Thus, the total timing of the RNAi and small molecule treatments are the same as in Figures 3 and 4. Cells were pre-extracted prior to fixation and microscopy analysis of the mean red fluorescence intensity per cell. Shown are representative images of DAPI and RNaseH^D10R-E48R^ (i.e., red fluorescence, scale bar = 10 μm) as well as the mean fluorescence intensity (MFI, red fluorescence) of 150 individual cells for each treatment (50 cells each from three independent experiments). Each graph represents images captured and analyzed with identical microscopy settings. Hence, the scale for each graph represents arbitrary units that are specific for the microscopy setting of that particular experiment. *P = 0.0001, †*P* = 0.02.

Finally, we sought to compare the effect of PRPF8 depletion and PlaB treatment on the integrity of RNA processing using cell biology assays. For one, loss of RNA processing has been linked to elevated levels of RNA/DNA hybrids (i.e. R-loops), and thereby cause genome instability [38]. Such R-loops can be resolved by RNAseH activity, such that a recent study used localization of a catalytically inactive form of RNaseH (D10R-E48R) as a proxy measurement for R-loop accumulation [39]. We used this approach rather than immunofluorescence with the S9.6 antibody [40], as we found it difficult to detect clear staining in U2OS cells. We generated a U2OS cell line with inducible expression of RNaseH^D10R-E48R^ fused to a nuclear localization signal and the mCherry fluorescent protein, as described [39]. Then, we induced expression of this fusion protein, and treated cells with an extraction buffer to remove non-chromatin bound protein, prior to fixation and imaging to quantify red fluorescence signal for individual cells. We found that treatment with siPRPF8-2, siPRPF8-4, and PlaB treatment each caused an increase in the mean red fluorescence intensity, compared to untreated controls (Figure 6). In contrast, treatment with the transcription elongation inhibitor DRB did not cause an increase in this signal (Figure 6). Thus, depletion of PRPF8 and PlaB treatment each cause an increase in chromatin-bound RNaseH^D10R-E48R^, which is consistent with an accumulation of R-loops.

**Figure 7:**
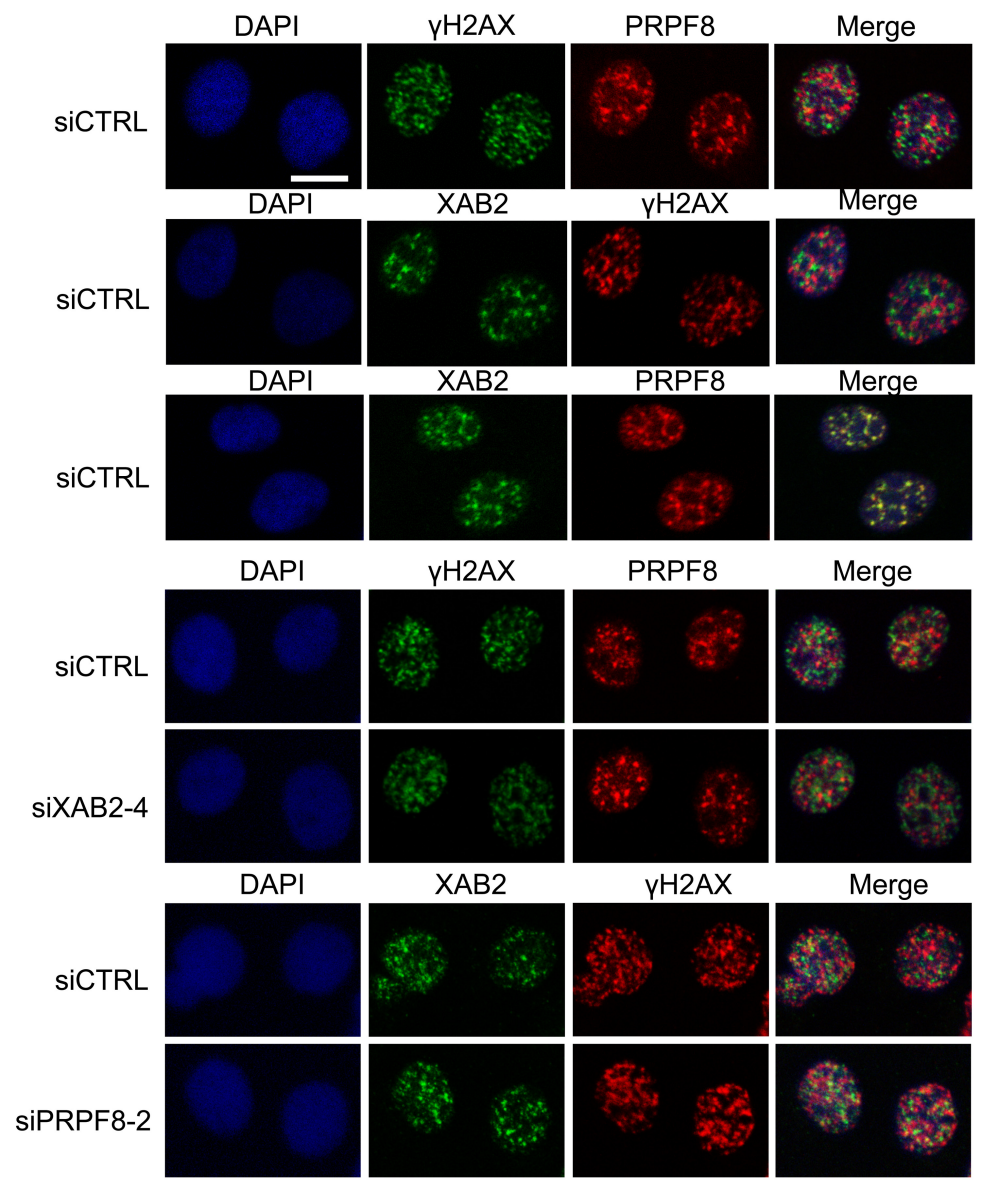
PRPF8 forms nuclear structures that co-localize with XAB2, which are consistent with interchromatin granules Cells were treated with the siRNAs shown, followed by 10 Gy IR and 30 min recovery prior to mild detergent pre-extraction, fixation, and immunofluorescence analysis. Shown are immunofluorescence signals from representative cells (scale bar = 10 μm).

**Figure 8:**
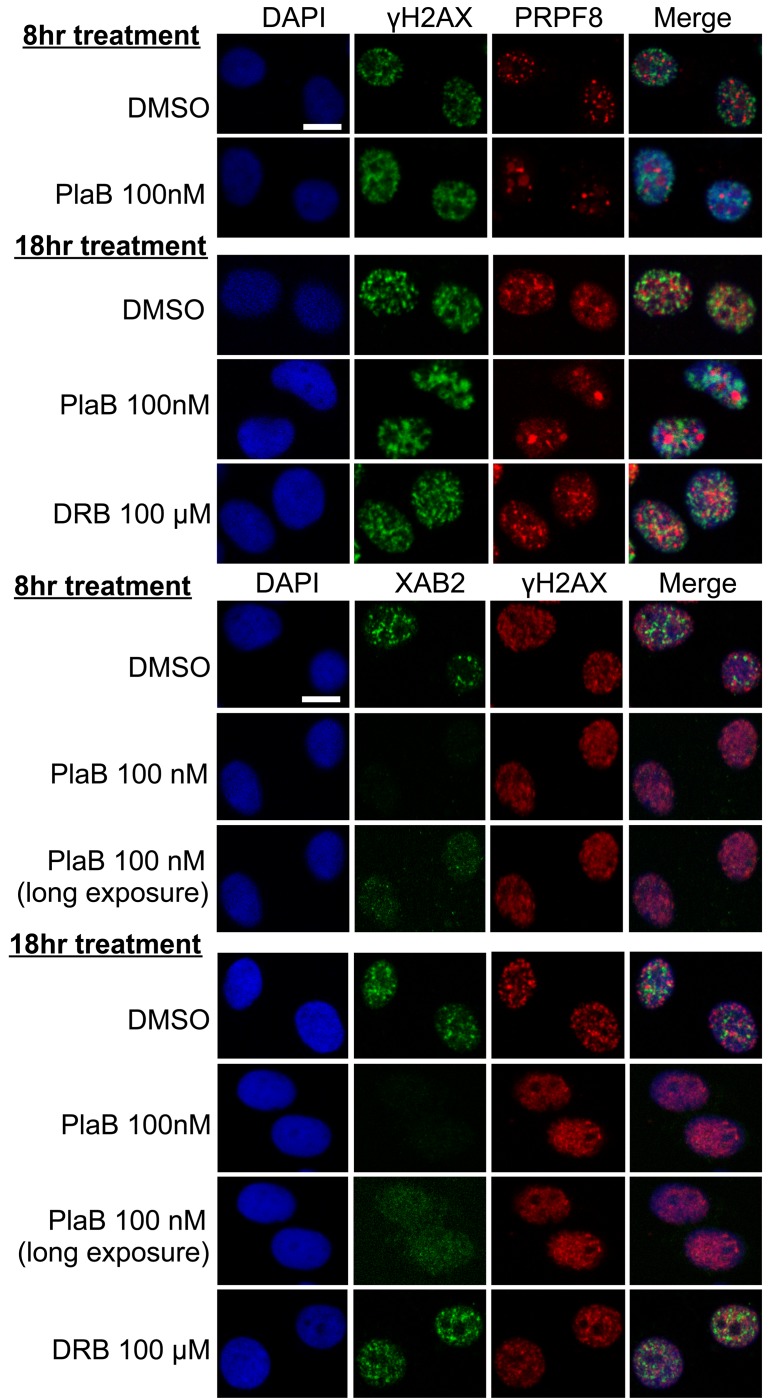
PlaB treatment causes PRPF8 to localize into mega-speckles, and disrupts XAB2 localization Cells were treated and analyzed as in Figure7, except including two different treatments of PlaB and DRB (8 hr and 18 hr total), during which the cells were also treated with 10 Gy IR treatment 30 minutes prior to pre-extraction and fixation. Shown are immunofluorescence signals from representative cells (scale bar = 10 μm).

We then examined the integrity of interchromatin granules, which are structures enriched in RNA processing factors [41]. Furthermore, XAB2 was previously shown to form interchromatin granule structures that are adjacent to DNA damage sites (i.e., γH2AX foci) [14]. We have repeated this XAB2 localization here, and find a similar localization for PRPF8, in that it forms nuclear structures that co-localize with XAB2, but are adjacent to γH2AX foci (Figure 7). We also examined these structures in cells treated with siXAB2 and siPRPF8, and found no obvious effect on PRPF8 localization in siXAB2-treated cells, nor on XAB2 localization in siPRPF8-treated cells (Figure 7). Similarly, DRB treatment did not obviously affect such localization (Figure 8). In contrast, PlaB treatment caused a substantial re-localization of PRPF8 into large structures (Figure 8), which have been referred to as mega-speckles [4, 42]. Furthermore, PlaB caused a marked disruption of XAB2 localization into diffuse staining (Figure 8). These findings indicate that PlaB treatment, but not PRPF8 depletion, causes a major disruption in interchromatin granule structures, which supports the notion that PlaB treatment causes a more general defect on nuclear function than PRPF8 depletion.

## DISCUSSION

**Figure 9:**
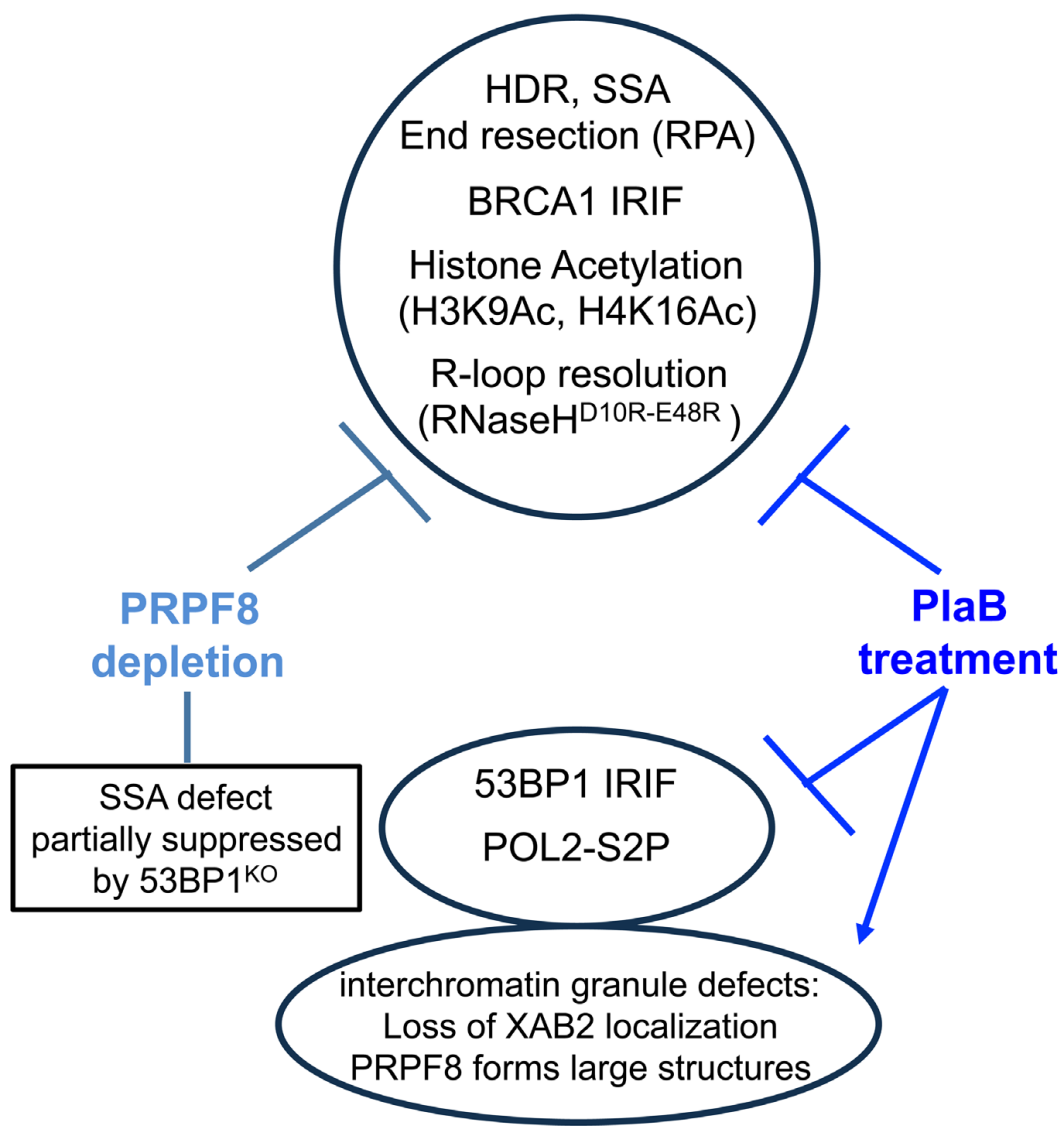
Shown is a summary of the effects of PRPF8 disruption and PlaB treatment on aspects of the DNA damage response and nuclear organization

To investigate the links between RNA splicing factors and genome stability, we have examined the effects of PRPF8 depletion and PlaB treatment on HR repair of chromosomal breaks (Figure 9). We found a common set of HR defects with these two treatments: a marked reduction in HDR and SSA sub-types of HR that require BRCA1, reduced end resection as measured by CPT-induced chromatin-bound RPA, and loss of BRCA1 foci. These HR defects are consistent with a loss of BRCA1 function during HR, and are similar to previous findings with XAB2 [14]. Furthermore, loss of 53BP1, which can rescue the SSA defect caused by BRCA1 depletion, can also partially suppress the SSA defect caused by PRPF8 depletion. However, in contrast, loss of 53BP1 had no effect on the SSA defect caused by PlaB treatment. Also, PlaB treatment caused a decrease in 53BP1 foci, and a marked reduction in BRCA1 expression. These latter findings are consistent with recent studies that PlaB treatment causes a reduction in HDR, BRCA1 expression, and both BRCA1 and 53BP1 foci [34]. We also found that PlaB treatment caused a disruption of PRPF8 and XAB2 localization in interchromatin granules. Altogether, these findings indicate that disrupting RNA splicing factors by depleting PRPF8 or PlaB treatment causes a loss of BRCA1 function during HR, but that PlaB treatment also causes a more general defect in the DNA damage response and nuclear organization (Figure 9).

These results have implications on targeting RNA splicing factors for cancer therapeutic approaches. Namely, we speculate that therapeutic targeting of splicing factors, including PRPF8 or XAB2, has the potential to disrupt BRCA1 function in cancer cells, which is known to correlate with improved therapeutic response to clastogens [43]. Furthermore, such disruption of BRCA1 function could be a targeted therapy for tumors with amplification of cyclin E, which is synthetically lethal with BRCA1 loss [44]. We also speculate that targeting XAB2 or PRPF8 may cause fewer general defects on nuclear function as SF3B inhibitors, such as PlaB and its derivatives, which were shown in clinical trials to cause substantial adverse events [2-5, 45]. Although, ongoing efforts to optimize small molecules that target SF3B may lead to anti-neoplastic agents that show less toxicity, which also could be used for individual tumors that may be particularly sensitive to SF3B inhibition [46-50]. Along these lines, screening tumors for deficiencies in XAB2, PRPF8, or other splicing factors could inform use of therapeutics that specifically target HR-deficient tumors, such as PARP inhibitors [43]. For example, since *PRPF8* mutations have been identified in myeloid malignancies [1, 2], therapeutics that target HR-deficiency may be worth considering for this patient population.

Regarding the role of PRPF8 in genome maintenance, it is important to note that this factor promotes both HDR and SSA, which have distinct mutagenic consequences. Namely, HDR has the potential to restore the original DNA sequence, but SSA always causes a deletion mutation between the repeat sequences used during repair [10]. Other factors implicated in end resection, including CtIP and BRCA1, also promote both HDR and SSA [10]. Accordingly, promoting end resection *per se* appears insufficient to specifically favor a non-mutagenic HR outcome. Thus, other aspects of the DNA damage response are required to regulate resection to bias repair towards HDR *vs*. SSA, such as the signaling pathway involving 53BP1 [10, 35, 51, 52].

The mechanism by which inhibition of splicing factors causes defects in BRCA1-mediated HR appears multifaceted, but importantly RNA splicing has significant effects on the chromatin states that facilitate HR. Namely, we find that PRPF8 depletion and PlaB treatment cause a marked reduction in H3K9Ac and H4K16Ac, but not H3K9me3. Such histone acetylation, particularly H4K16Ac, has been shown to be important for recruitment of BRCA1 to DNA damage, as well as homologous recombination [27, 36, 37]. In addition, inhibition of RNA splicing can disrupt transcription unit function, which we speculate could compete for BRCA1 activity, and thereby diminish the response of BRCA1 to DNA damage. Consistent with this model, BRCA1 has also been shown to be important for resolution of R-loops [53, 54], which can be caused by inhibition of RNA splicing factors, as demonstrated in several reports [7, 12, 38]. Indeed, we have found that PRPF8 depletion and PlaB treatment cause an accumulation of chromatin bound RNaseH^D10R-E48R^, which is a proxy measurement for R-loop accumulation [39]. Also in support of a competition model for BRCA1 function, BRCA1 can associate with RNA splicing complexes [20], although, we were not able to reproduce this association of PRPF8 and BRCA1 in our study. Nevertheless, we suggest that the changes to chromatin and transcription unit function caused by RNA splicing inhibition contribute to the defects in BRCA1-mediated HR.

Related to the above models, a recent study suggested that loss of ubiquitin signaling at DNA damage sites, specifically a reduction in RNF8, may be a major contributor to the HR defects caused by inhibition of RNA splicing. Their finding that PlaB treatment causes a reduction in both BRCA1 and 53BP1 foci [34], which we confirmed here, is consistent with their model, because RNF8 promotes focal accumulation of these factors [31-33]. However, RNF8 is dispensable for HDR and SSA [55]. Indeed, similar to loss of 53BP1, loss of RNF8 can partially rescue the HR defects caused by BRCA1 depletion [55]. Accordingly, loss of RNF8 cannot readily explain the marked defect in HDR and SSA caused by PlaB treatment, although it could contribute to the reduction in BRCA1 and 53BP1 foci.

In contrast to these models, PRPF8 and other RNA splicing factors could also have a direct role in repair at sites of DNA damage, or conversely could function largely through shaping the transcriptome in such a manner as to specifically disrupt BRCA1-mediated HR. Regarding the former model, PRPF8 was identified in a complex with the single-stranded binding complex RPA, along with another spliceosome-associated factor PRP19, which has been shown to be recruited to DNA damage and promote ubiquitination of RPA [15-18]. It is conceivable that PRPF8 could function in this process, although we have found a strong adjacent localization of PRPF8 with sites of DNA damage (γH2AX), which is inconsistent with a direct role at sites of DNA damage. Regarding effects on the transcriptome, of course the marked reduction in BRCA1 expression caused by PlaB treatment likely contributes to loss of BRCA1 function during HR. In contrast, we did not observe an obvious reduction in BRCA1 levels in PRPF8 depleted cells. Furthermore, transcriptome studies of cells depleted of PRPF8 and XAB2 have not revealed obvious gene expression changes that could explain defects in BRCA1-mediated HR [56, 57]. In contrast, such studies have found that proper expression of mitotic progression genes is particularly sensitive to depletion of PRPF8 and XAB2, causing defects in anaphase [56, 57]. Although, notably disrupting HR can cause similar anaphase defects [58, 59]. In summary, while the influence of RNA splicing on genome stability appears to have many layers of complexity, we suggest that a key role of this process is to support a chromatin state that is permissive for BRCA1 function during HR.

## MATERIALS AND METHODS

### Cell lines, siRNA, plasmids, and small molecules

Establishment and culturing of U2OS reporter cell lines (derived from a line directly obtained from ATCC), and the expression vectors for I-SceI (pCBASce) and 3xflag-XAB2 (3xf-XAB2) were each described previously [14, 60, 61]. The U2OS SA-GFP reporter cell line was used for all of the immunoblotting and cell biology analysis. The 53BP^KO^ cell line was derived from the U2OS SA-GFP cell line by transient expression of CAS9 and two single guide RNAs (5’gCATAATTTATCATCCACGTC, 5’gAGAGAATGAGGCTCGAAGTG) cloned separately into px330 (Addgene 42230) [62], along with the dsRED expression vector (Clontech). Following sorting for dsRED+ cells, individual clones were screened by immunoblotting to identify the 53BP^KO^ cell line. The inducible RNaseH^D10R-E48R^ U2OS cell line was derived by transfecting the pICE-RNaseHI-D10R-E48R-NLS-mCherry plasmid (Addgene #60367) [39] with the pCDNA6/TR regulatory plasmid (Thermofisher), and selecting clones in 5 μg/ml blasticidin, which were screened for doxycycline-inducible red fluorescence.

The pCAGGS-I-SceI-GR plasmid was generating by fusing the glucocorticoid receptor ligand binding domain (GR) coding sequence amplified from I-SceI-GR-RFP (gift of Dr. Tom Misteli) [25] downstream from pCAGGS-I-SceI [61]. The pCAGGS-myc-PRPF8 expression vector was derived from Origene clone SC116070, and generated with silent mutations at the siPRPF8-2 site, 5’ GCcGAcGGgcTaCAGTAcA. Sequences of siRNAs (GE/Dharmacon) are: non-targeting siCTRL (D-001810-01) 5’-ugguuuacaugucgacuaa, siPRPF8-2 (D-012252-02) 5’gcagauggauugcaguaua, siPRPF8-4 (D-012252-04) 5’ggaagaagcuaacuaaugc, siXAB2-4 (D-004914-04), 5’-ccaauucucugucaaaugc, siBRCA1 (D-003461-06) 5’gggauaccaugcaacauaa.

Pladienolide B (PlaB, Santa Cruz Biotechnology, sc391691) was dissolved in Dimethyl Sulfoxide (DMSO): to a stock concentration of 100 µM, and 5,6-Dichlorobenzimidazole 1-β-D-ribofuranoside (DRB, Sigma D1916) was resuspended at a concentration of 50 mM in DMSO. Cells were treated with PlaB at 100 nM, and DRB at 100 µM. All treatments with PlaB and DRB included a prior treatment with siCTRL to enable direct comparison with the RNAi experiments.

### DSB reporter assays

Cells were treated with siRNA by seeding 0.5-1 x 10^5^ U2OS cells on a 24 well plate in 0.6 ml antibiotic-free media with 5 pmol of siRNA that had been incubated with 1.8 μl RNAiMAX (Invitrogen/Thermofisher). Following the overnight siRNA treatment, cells were transfected with 0.3 μg of the I-SceI expression vector (pCBASce) along with either 0.3 μg of empty vector, or myc-PRPF8 expression vector, using 1.8 μl Lipofectamine 2000 (Invitrogen/Thermofisher), in 0.6 ml antibiotic-free media. The transfection media was removed after 3 hrs, and replaced with antibiotic media. For the PlaB and DRB experiments, 0.4 μg of I-SceI-GR expression vector was used, and the day after transfection, cells were pre-treated (2 hr) with the relevant small molecules prior to treatment that included the I-SceI-GR inducing agent (Triamcinolone Acetonide, TA, 100 nM). Three days after the plasmid transfections, GFP+ frequencies were determined by flow cytometery using a CyAn ADP Analyzer (Beckman Coulter, Inc.), as described previously [60]. The GFP+ frequency for each transfection was divided by the mean value for the control samples treated in parallel (i.e., siCTRL+EV, or siCTRL+DMSO). Each repair value is the mean of multiple independent transfections, error bars reflect the standard deviation, and statistics were performed with the unpaired *t*-test.

### Microscopy analysis

For ionizing radiation induced foci analysis, siRNA treatment was performed as described for the DSB reporter assays, and subsequently cells were plated onto chamber slides, which were treated with 10 Gy of IR (Gammacell 3000) and allowed to recover for 6 hr prior to fixation. For localization studies of PRPF8 and XAB2, cells were treated with 10 Gy of IR, but allowed to recover for 30 min, and were treated with pre-extraction buffer (20 mM HEPES, 50 mM NaCl, 1 mM EDTA, 3 mM MgCl2, 300 mM sucrose, 0.25% Triton-X 100) just prior to fixation. Slides were fixed with 4% paraformaldehyde and treated with 0.1 M glycine and 0.5% Triton-X 100 prior to probing with antibodies against PRPF8 (Abcam ab79237, or Bethyl A303-921A), BRCA1 (Santa Cruz sc6954), γH2AX (active motif 39117, or Novus NB100-78356), 53BP1 (Abcam ab36823), XAB2 (HCNP, Santa Cruz Biotech sc-271037), and followed by secondary antibodies (Life Technologies, A-11036 and A-11029), and with DAPI using Vectashield Mounting Medium (Vector Laboratories H1500). Images were acquired using a BX-50 (Olympus) microscope at 40X magnification with Image-Pro software. Confocal microscopy images were acquired at 40X magnification using the Zeiss LSM 700 Confocal Microscope, using the ZEN Black image acquisition software. For three independent treatments per condition, 50 cells were scored for those with ≥10 foci. Statistics were performed as for the reporter assays.

For detection of chromatin-bound RNAseH-D10R-E48R-NLS-mCherry, the inducible RNaseH^D10R-E48R^ U2OS cells were transfected with siRNA for 20 hours before being plated to chamber slides. Cells were treated with 1µg/ml doxycycline (DOX, Sigma D9891) for 24 hours before fixation. In small molecule treated cells, the cells were pre-treated with 1µg/ml DOX for 6 hours followed by 18 hours PlaB (100 nM) or DRB (100 µM) treatment in the presence of DOX. Cells were pre-extracted and fixed as described above for PRPF8 localization, and mounted with Vectashield Mounting Medium. Images were captured using an Olympus BX-50 microscope at 40X magnification with Image-Pro and HCImage software. Intensity of RNAseH-D10R-E48R-NLS-mCherry signal was analyzed using Image-Pro Premier 9.1 software. 150 cells from three independent experiments were scored. Statistical tests were performed by GraphPad Prism 7.03, using a one-way ANOVA and a Dunnet’s multiple comparisons test.

### Immunoblotting analysis and immunoprecipitation

To generate protein extracts, cells were lysed in ELB buffer: 50 mM HEPES, 250 mM NaCl, 5 mM EDTA, 0.1% IGEPAL, Protease Inhibitor Cocktail (Roche 11697498001). Lysates were sonicated (QSonica Q800RS ultrasonic horn), and soluble material collected by centrifugation. Alternatively, cells were lysed with NETN (20 mM Tris pH 8, 100 mM NaCl, 1 mM EDTA, 0.5% IGEPAL, 1.25 mM DTT and Roche Protease Inhibitor), using several freeze/thaw cycles. To examine histone modifications and POL2-S2P levels, cells were pre-extracted using Triton-buffer (25mM Hepes pH 7.4, 50mM NaCl, 1mM EDTA, 3mM MgCl2, 300mM Sucrose, 0.5% Triton X-100) with 10 mM sodium butyrate, scraped into SDS loading buffer (62.5 mM Tris-HCl pH 6.8, 2% SDS, 10% glycerol, 0.01% bromophenol blue, and 140 mM DTT), and then boiled, sonicated (QSonica Q800RS ultrasonic horn), and boiled again.

For PRPF8 and BRCA1 co-IP analysis, 5x10^6^ U2OS cells were plated on 10 cm plates. Subsequently, cells were treated with 10 Gy of IR and incubated for 2 hours at 37°C. Cells were lysed for 3 hours at 4°C in ELB buffer, with the addition of phosSTOP (Roche 04906845001) and 30 units per ml of benzonase (Sigma-Aldrich E1014). For XAB2 co-IP analysis, 1x10^6^ U2OS cells were transfected with 5 μg 3xf-XAB2 expression vector or EV and 15 μl Lipofectamine 2000 in 3 ml. Cells were lysed in IP buffer: 20mM Tris pH 8.0, 150 mM NaCl, 1mM EDTA, 0.5% IGEPAL, phosSTOP, Protease Inhibitor Cocktail with 30 units per ml of benzonase. Lysates for the 3xf-XAB2 IP were homogenized with a dounce, and those for the PRPF8 and BRCA1 IPs were sonicated (QSonica Q800RS ultrasonic horn). Soluble material was pre-cleared with protein-G Dynabeads (Novex 10003D) prior to incubation with 2 μg of PRPF8 antibody (Bethyl A303-921A-T), BRCA1 (Millipore 07-434), Flag antibody (Sigma catalog F3165), or IgG rabbit antibody (Abcam 27478), followed by addition of protein-G Dynabeads, which were washed with IP buffer, eluted with 100 mM Glycine pH 2.5, and neutralized with 1 M Tris-HCl pH 10.85.

Blots of these extracts or IPs were probed with antibodies described above for the microscopy analysis, as well as H4K16Ac (Epigentek A4030), H3K9Ac (Upstate 06-942), H3K9me3 (Upstate 07-442), POL2-S2P (Abcam ab5095), Actin (Sigma A2066), and HRP-conjugated secondary antibodies (Santa Cruz Biotech, sc-2004, sc-2005, sc-2020). ECL reagent (Amersham Biosciences) was used to develop HRP signals.

### Cell cycle and end resection analysis

To examine cell cycle profiles, after siRNA treatment, cells were incubated with 10 μM Bromodeoxyuridine (BrdU) for 30 minutes prior to fixation, and staining with both FITC-labeled anti-BrdU antibody (BD Pharmingen 51-33284X), and propidium iodide (PI). The end resection assay was performed as previously described [29, 63]. Briefly, cells were treated with 1 μM camptothecin (CPT) in media for 1 hr, collected by trypsinization, washed with PBS, treated with 0.2% Triton X-100 in PBS, fixed with BD cytofix/cytoperm buffer, stained with RPA34 antibody (antibody 9H8, Abcam ab2175), or γH2AX antibody (Millipore 05-636), followed by secondary staining with Alexa Fluor 488 goat anti-mouse (Life Technologies A-11029). Cells were counterstained with DAPI (Sigma, R4642). Staining for both assays was analyzed with a CyAn ADP Analyzer (Beckman Coulter, Inc.) flow cytometer.

### qRT-PCR

RNA was isolated from treatments as performed for the end resection analysis using the RNeasy Plus Minikit (Qiagen 74134). The RNA was treated with M-MLV Reverse Transcriptase (Promega M170A) to generate cDNA, which was amplified in a Applied Biosystems 7500 Fast Real Time PCR system using SYBR-green (Applied Biosystems 4472908), with primers for Actin 5’-ACTGGGACGACATGGAGAAG and 5’-AGGAAGGAAGGCTGGAGGAG, or BRCA1 5’-TGACTCTGGGGCTCTGTCTT and 5’-GCATCTGGGTGTGAGAGTGA.

## SUPPLEMENTARY MATERIALS FIGURE



## Abbreviations

BrdU: Bromodeoxyuridine
CPT: Camptothecin
DMSO: Dimethyl Sulfoxide
DOX: Doxycycline
DRB: 5,6-Dichlorobenzimidazole 1-β-D-ribofuranoside
DSB: Double Strand Break
EJ: End-joining
GFP: Green Fluorescent Protein
HDR: Homology-Directed Repair
HR: Homologous Recombination
PI: propidium iodide
PlaB: Pladienolide B
POL2-S2P: RNA polymerase II, phosphorylated at the serine 2 position of the C-terminal domain
RPA: Replication Protein A
SSA: Single Strand Annealing
TA: Triamcinolone Acetonide
