# Supplementary material for: PRPF8 is important for BRCA1-mediated homologous recombination
Source: Oncotarget. 2017 Oct 6;8(55):93319–37. doi: 10.18632/oncotarget.21555 (PMC5706798; doi:10.18632/oncotarget.21555)
Supplement: Supplementary file 1 [file oncotarget-08-93319-s001.pdf]

# PRPF8 is important for BRCA1-mediated homologous recombination

## SUPPLEMENTARY MATERIALS

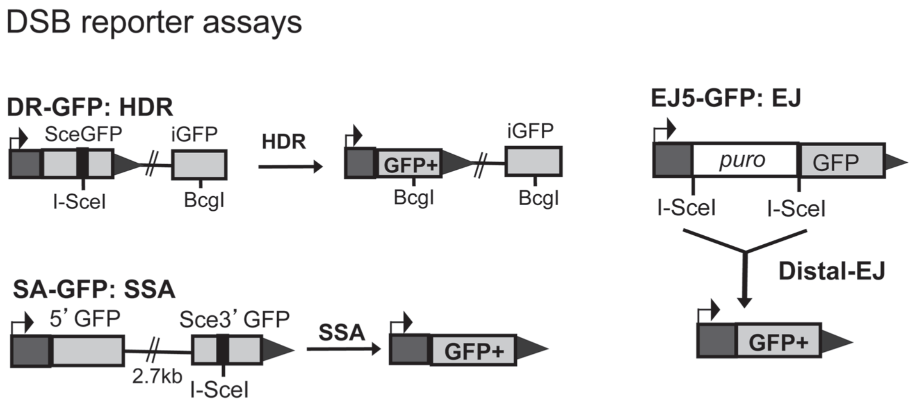


**Supplementary Figure 1: Shown are diagrams of the DR-GFP, SA-GFP, and EJ5-GFP reporter assays.**
